# Spectral Response Based Calibration Method of Tristimulus Colorimeters

**DOI:** 10.6028/jres.103.040

**Published:** 1998-12-01

**Authors:** George Eppeldauer

**Affiliations:** National Institute of Standards and Technology, Gaithersburg, MD 20899-0001

**Keywords:** chromaticity coordinates, color calibration, color temperature, colorimetry, detector standard, photometry, spectral response, tristimulus values

## Abstract

A new method is described to calibrate tristimulus colorimeters for high accuracy color measurements. Instead of traditional lamp standards, modern, high accuracy detector standards are suggested for calibration. After high accuracy absolute spectral response determination of the tristimulus receivers, color (spectral) correction and peak (amplitude) normalization can minimize uncertainties caused by imperfect realizations of the Commission Internationale de l’Eclairage (CIE) color matching functions. As a result of the corrections, stable light sources of different spectral power distributions can be measured with an accuracy dominated by the sub tenths of a percent uncertainty of novel spectral response determinations.

## 1. Introduction

Tristimulus colorimetry is based on light measurement using three or more receivers with spectral responsivities matched to the Commission Internationale de l’Eclairage (CIE) $\bar{x}(\lambda )$, $\bar{y}(\lambda )$, and $\bar{z}(\lambda )$ color matching functions [1]. To achieve accurate measurements for a large variety of light sources, the spectral matches should be as close as possible to the color matching functions. The receivers are usually realized with silicon photodiodes and attached filter packages [2]. Usually, the spectral mismatch between the realized and the color matching functions give the dominant uncertainties in tristimulus color measurements.

At present, tristimulus colorimeters are calibrated with standard lamps. The calibration of the most frequently used color temperature standard lamps is derived from source-based spectral irradiance scales. NIST reported a 0.67 % relative expanded uncertainty (*k* = 2)^1^ for the disseminated spectral irradiance standard lamps in the visible range and a relative 0.59 % long-term reproducibility [3]. Research is being conducted at NIST to decrease the 0.67 % uncertainty by a factor of three still using standard lamps [4]. The accuracy of the standard lamp influences the photometric [receiver matched to $\bar{y}(\lambda )$] accuracy of the tristimulus colorimeter. The wavelength dependent (e.g., burning time caused) changes of the standard lamp influence the colorimetric accuracy of the tristimulus meter.

Several commercially available colorimeters were compared for accuracy by measuring nine different laser lines (saturated colors) by Berns et al. [6]. The differences between the theoretical and the measured chromaticity coordinates were reported. The lowest rms errors in *x* and *y* (0.0042 and 0.0051, respectively) were observed on a tristimulus colorimeter employing partial (mosaic) filters. The errors are expected to decrease to a large extent for white light sources, such as tungsten lamps. The reported errors were dominated by spectral response deviations (mismatch) of the receivers relative to the CIE recommended color matching functions. The uncertainties of the calibrating standard lamps also contributed to the results.

In contrast to source standards, primary standard detectors (cryogenic radiometers) can measure optical (radiant) power with an uncertainty of 10^−4^ [7, 8]. Certain type silicon detectors, in a light-trap arrangement, can be calibrated against primary standard radiometers using several intensity stabilized lasers [9]. With interpolation between the laser lines, a 0.06 % uncertainty in spectral responsivity was reported on light-trap Si detectors between 406 nm and 920 nm [10]. This uncertainty is about one fourth of the uncertainties reported for traditional monochromator based detector spectral response measurements [11].

Achievable total uncertainty of spectral transmittance measurements on high quality color filters is reported to be 2 × 10^−4^ [12].

A narrow-band filter-radiometer was calibrated for spectral irradiance response in the visible region against a trap detector with an uncertainty of 0.07 % [13] for measured irradiance. In that report, the dominant uncertainty components were the uncertainties of the trap detector response, 0.036 %, the area of the trap aperture, 0.034 %, and the wavelength reproducibility, 0.026 %.

If the uncertainties of the receiver response measurements are very small, the spatial response non-uniformities of the filter-detector packages could limit the accuracy of the tristimulus measurements. This problem can be avoided if apertures are used in front of the filter-detector packages and the apertures are overfilled with the uniform field of the (point) source to be measured. The area of the apertures can be measured with an uncertainty of 0.026 % [14].

The motivation behind the development of a detector-based calibration method for tristimulus colorimeters was to utilize the significantly lower uncertainty of new detector standards compared with traditional lamp standards. The goal of the method described in this paper is to determine broad-band calibration factors for all receivers in a tristimulus colorimeter to minimize measurement uncertainty. If the spectral response determination of the receivers is accurate, application of the calibration factors for different source distributions will result in high colorimetric accuracy. This multiple receiver method is an extension of the single receiver spectral mismatch correction used in our detector-based illuminance scale realization [15, 16].

## 2. Theoretical Basis

In order to determine *x*, *y* chromaticity coordinates of a light source, the CIE tristimulus values of the source are to be obtained by

$$\begin{array}{l} X=k\int_{\lambda }S(\lambda )\;\bar{x}(\lambda )\;d\lambda \\ Y=k\int_{\lambda }S(\lambda )\;\bar{y}(\lambda )\;d\lambda \\ Z=k\int_{\lambda }S(\lambda )\;\bar{z}(\lambda )\;d\lambda , \end{array}$$ (1)

where *S*(*λ*) is the spectral power distribution of the source to be measured; $\bar{x}(\lambda )$, $\bar{y}(\lambda )$, and $\bar{z}(\lambda )$ are the 1931 CIE color-matching functions; and *k* is a normalization factor. In practice, $\bar{x}(\lambda )$ is realized by two receivers, ${\bar{x}}_{L}(\lambda )$ and ${\bar{x}}_{S}(\lambda )$:

${\bar{x}}_{S}(\lambda )=0$ and ${\bar{x}}_{L}(\lambda )=\bar{x}(\lambda )$ if the wavelength is longer than 504 nm, and
${\bar{x}}_{L}(\lambda )=0$ and ${\bar{x}}_{S}(\lambda )=\bar{x}(\lambda )$ if the wavelength is shorter than 504 nm.

(Note that the subscripts L and S mean long and short, respectively.)

The CIE tristimulus value *X* can be written as:

$$X=X_{1}+X_{2},$$ (2)

where

$$\begin{array}{l} X_{1}=k\int_{\lambda }S(\lambda )\;{\bar{x}}_{L}(\lambda )\;d\lambda ,\;\text{and}\\ X_{2}=k\int_{\lambda }S(\lambda )\;{\bar{x}}_{S}(\lambda )\;d\lambda . \end{array}$$

*Y* in Eq. (1) will give an absolute photometric quantity (e.g., in lux) [15] if

$$k=K_{m}=683\;\mathrm{lm}/W.$$ (3)

The measured photodiode output currents of the four separate receivers are

$$\begin{array}{l} I_{X1}=\int_{\lambda }S(\lambda )\;s_{XL}(\lambda )\;d\lambda \\ I_{X2}=\int_{\lambda }S(\lambda )\;s_{XS}(\lambda )\;d\lambda \\ I_{Y}=\int_{\lambda }S(\lambda )\;s_{Y}(\lambda )\;d\lambda \\ I_{Z}=\int_{\lambda }S(\lambda )\;s_{Z}(\lambda )\;d\lambda . \end{array}$$ (4)

where *s_X_*_L_(*λ*), *s_X_*_S_(*λ*), *s_Y_*(*λ*), and *s_Z_*(*λ*) are the absolute spectral responsivities of the receivers.

When measuring a light source of known spectral power distribution *S*(*λ*), the receiver calibration factors can be determined from the ratio of Eq. (1) to Eq. (4):

$$\begin{array}{l} k_{X1}=\frac{X_{1}}{I_{X1}}=\frac{K_{m}\int_{\lambda }S(\lambda )\;{\bar{x}}_{L}(\lambda )\;d\lambda }{\int_{\lambda }S(\lambda )\;s_{XL}(\lambda )\;d\lambda }\\ k_{X2}=\frac{X_{2}}{I_{X2}}=\frac{K_{m}\int_{\lambda }S(\lambda )\;{\bar{x}}_{S}(\lambda )\;d\lambda }{\int_{\lambda }S(\lambda )\;s_{XS}(\lambda )\;d\lambda }\\ k_{Y}=\frac{Y}{I_{Y}}=\frac{K_{m}\int_{\lambda }S(\lambda )\;\bar{y}(\lambda )\;d\lambda }{\int_{\lambda }S(\lambda )\;s_{Y}(\lambda )\;d\lambda }\\ k_{Z}=\frac{Z}{I_{Z}}=\frac{K_{m}\int_{\lambda }S(\lambda )\;\bar{z}(\lambda )\;d\lambda }{\int_{\lambda }S(\lambda )\;s_{Z}(\lambda )\;d\lambda }. \end{array}$$ (5)

By *normalizing* the color matching functions to their peak values, the receiver calibration factors can be written as:

$$\begin{array}{l} k_{X1}=\frac{1.06291\;K_{m}\;F_{X1}}{s_{XL}(599)}\\ k_{X2}=\frac{0.3501\;K_{m}\;F_{X2}}{s_{XS}(442)}\\ k_{Y}=\frac{K_{m}\;F_{Y}}{s_{Y}(555)}\\ k_{Z}=\frac{1.78297\;K_{m}\;F_{Z}}{s_{Z}(446)} \end{array}$$ (6)

by introducing the color correction factors:

$$\begin{array}{l} F_{X1}=\frac{\int_{\lambda }S(\lambda )\;{\bar{x}}_{\mathrm{Ln}}(\lambda )\;d\lambda }{\int_{\lambda }S(\lambda )\;s_{X\mathrm{Ln}}(\lambda )\;d\lambda }\\ F_{X2}=\frac{\int_{\lambda }S(\lambda )\;{\bar{x}}_{\mathrm{Sn}}(\lambda )\;d\lambda }{\int_{\lambda }S(\lambda )\;s_{X\mathrm{Sn}}(\lambda )\;d\lambda }\\ F_{Y}=\frac{\int_{\lambda }S(\lambda )\;V(\lambda )\;d\lambda }{\int_{\lambda }S(\lambda )\;s_{Yn}(\lambda )\;d\lambda }\\ F_{Z}=\frac{\int_{\lambda }S(\lambda )\;{\bar{z}}_{n}(\lambda )\;d\lambda }{\int_{\lambda }S(\lambda )\;s_{Zn}(\lambda )\;d\lambda }, \end{array}$$ (7)

where *s*_XL_(599), *s_X_*_S_(442), *s_Y_*(555), and *s_Z_*(446) are the absolute responses of the realized receivers at the peak wavelengths of the color matching functions; and *s_X_*_Ln_(*λ*), *s_X_*_Sn_(*λ*), *s_Y_*_n_(*λ*), and *s_Z_*_n_(*λ*) are the relative responses of the realized receivers normalized also at the peak wavelengths of the color matching functions. The peak wavelengths of the realized receivers are not necessarily equal to the peak wavelengths of the color matching functions.

A color correction factor will be unity if the normalized channel response is equal to the normalized CIE color matching function.

Once the tristimulus colorimeter is calibrated for *k_X_*_1_, *k_X_*_2_, *k_Y_*, and *k_Z_*, the tristimulus values of a test light source can be measured as

$$\begin{array}{l} X^{\prime }=X_{1}\prime +X_{2}\prime \;\text{where}\;X_{1}\prime =k_{X1}I_{X1}\prime \;\text{and}\;X_{2}\prime =k_{X2}\;I_{X2}\prime \\ Y^{\prime }=k_{Y}\;I_{Y}\prime \\ Z^{\prime }=k_{Z}\;I_{Z}\prime \end{array}$$ (8)

where *I_X_*_l_′, *I_X_*_2_′, *I_Y_*′, and *I_Z_*′ are the measured output currents of the receivers.

The calibration procedure can be applied to various measurement geometries (e.g., illuminance, luminance, luminous flux, or luminous intensity) depending on the units in which *s_X_*_L_(599), *s_X_*_S_(442), *s_Y_*(555), and *s_Z_*(446) are expressed.

## 3. Achievable Accuracy

In order to obtain the highest color measurement accuracy, the receiver calibration factors are to be redetermined for all *S*(*λ*) source distributions to be measured. The spectral mismatch of the receivers, relative to the CIE functions, should be small to allow for relatively large uncertainties when determining *S*(*λ*). It was shown in an earlier work [16] that the change of *F_Y_* with a high quality spectral match of *f*_1_′ = 1.43 % [2], was 0.1 % for a color temperature change from 2600 K to 3200 K of a Planckian radiator. With a lower quality spectral match of *f*_1_′ = 3.4 %, which is typical for the red and blue receivers, the change in *F_Y_* would be larger, still allowing for a large enough uncertainty of *S*(*λ*). The final *S*(*λ*) for tungsten lamps, which are more or less similar to Planckian radiators [17], can be obtained by iterating the Planckian function (at different temperatures) and the tristimulus measurements, until the highest color measurement accuracy is reached. For other types of sources with smoothly varying spectral power distribution (e.g., many kinds of paints, color tiles, etc.), *S*(*λ*) can be measured with a low accuracy spectroradiometer, and the color measurement accuracy still remains high.

According to the references in the Introduction, the presently achievable relative expanded uncertainty of absolute spectral response determinations is on the order of 0.1 %. The uncertainty of relative spectral response measurements can be lower because of the smaller number of uncertainty components. The uncertainties of the tristimulus values propagate to the uncertainties of the chromaticity coordinates. The chromaticity coordinates can be calculated from the tristimulus values via

$$x=\frac{X}{X+Y+Z},\;y=\frac{Y}{X+Y+Z}.$$ (9)

Now, assume that the relative uncertainties of *X*, *Y*, and *Z* are all 0.1 %. For a Standard Illuminant A, where *x* = 0.4476 and *y* = 0.4074, the worst case scenario (Δ*X* = + 0.1 %, Δ*Y* = 2 0.1 %, and Δ*Z* = 0 %) shows a chromaticity coordinate change of ≅0.0004 for both *x* and *y*, resulting in a ≅10 K change in the correlated color temperature. These expanded uncertainties are similar to those reported for the source-based NIST color temperature scale [18]. With improvements of spectral response determinations (as suggested below), the accuracy of detector-based tristimulus color measurements can be further increased.

## 4. Suggested Realizations

The described detector-based calibration method can be applied to the calibration of existing tristimulus colorimeters where the spectral response of the receivers can be measured. The achievable color measurement accuracy will depend on the uncertainty of the spectral response measurements.

Standard quality tristimulus colorimeters can be constructed using high accuracy detector standards such as trap detectors. The advantage of using silicon trap detector standards is that the spectral response of the trap detector can be determined directly against a primary standard cryogenic radiometer [10]. Further advantages of trap detectors are relative response non-uniformities of less than 0.02 % [19] and very low reflectance in the visible wavelength range [9], especially for transmission type versions [20, 21]. The filter packages can be measured separately if they are used with transmission-type trap detectors because of zero back reflection from these detectors.

We have started developing a new facility to calibrate illuminance measuring photometers and tristimulus colorimeters against irradiance measuring trap detectors. These trap detectors will be calibrated against the cryogenic radiometer in the power (detector is underfilled by the laser beam) measurement mode. The trap detectors are equipped with precision apertures and will measure the well-collimated radiation (within the aperture) from point sources to obtain the highest accuracy. The point sources are being realized with small integrating spheres illuminated by tunable lasers. The aperture areas are also measured with point sources. The accuracy of our cryogenic radiometer is being improved to achieve radiant power measurements with an uncertainty of between 0.01 % and 0.02 %. The expected spectral irradiance response uncertainty of the irradiance trap detectors (for point sources) is about 0.03 %. All of our existing illuminance-type meters can be calibrated against the irradiance trap detectors with substitution in the uniform field of the tunable monochromatic point source. After spectral response calibrations, the described correction method will be applied. A tristimulus colorimeter calibrated this way will have a chromaticity coordinate measurement uncertainty (expanded but not relative) of about 0.0003. The accuracy of matrix corrected tristimulus colorimeters [22, 23, 24] can also be improved if they are calibrated against the suggested response corrected reference tristimulus colorimeters. Further analysis is suggested to establish a relationship between the *f*_1_′ of the receivers and the colorimetric accuracy when spectrally structured and changing source spectral power distributions (e.g., color TV monitors) are measured.

## 5. Conclusion

The significant decrease of uncertainties in detector spectral response measurements in the past 5 years motivated the development of a detector-based calibration method for tristimulus colorimeters.

A method for color (spectral mismatch) correction and peak (amplitude) normalization of color measuring tristimulus receivers has been developed. Broad-band calibration factors, based on spectral response measurements of the receivers, can be determined to minimize the color measurement uncertainties caused by the imperfect receiver response realizations. The method utilizes the lower uncertainty of new detector standards relative to traditional lamp standards. The calibration factors can be determined for sources of different spectral power distributions.

Application of the described detector-based calibration method will result in chromaticity coordinate expanded uncertainties (not relative) of less than 0.001. This corresponds to a color temperature measurement accuracy equal to or better than that of presently used primary lamp standards.

## Footnotes

1 Throughout this paper uncertainties are given as relative expanded uncertainties with a coverage factor *k* = 2 unless otherwise stated [5].
